# Lymphatic Filariasis in Soldiers Exposed in INDOPACOM

**Published:** 2024-08-20

**Authors:** G. Dennis Shanks, James K. Smith

**Affiliations:** 1 Australian Defence Force Malaria and Infectious Disease Institute, Enoggera, Queensland; 2 University of Queensland, School of Public Health, Brisbane, Australia; 3 Metro North Hospital and Health Service, Queensland Health, Herston, Queensland, Australia

## Abstract

Some military organizations in the U.S. Indo-Pacific Command (INDOPACOM) give returning soldiers presumptive treatment for filariasis. As there have been few clinical cases in recent decades, the historical basis for this chemotherapy was reviewed. During the Second World War, U.S. Marines stationed on Polynesian islands such as Tonga, Samoa, and Fiji experienced clinical lymphatic filariasis. Although thousands of both U.S. and Australian soldiers served in New Guinea, few, if any, cases of lymphatic filariasis were ascribed to Melanesia. While the French Army reported dozens of cases of filariasis among its service members during the 1950s Vietnam conflict, the U.S. military experienced only a few cases among the nearly 2 million service members who served in Vietnam in the 1960s. Australian soldiers deployed to Timor Leste in the 21st century showed rare seroconversions to filaria but no clinical disease. Following mass drug administration to eliminate lymphatic filaria in the INDOPACOM region, exposure in deployed soldiers rarely occurs and preventive chemotherapy should cease.

*“No specific therapy was employed because none of the men were ever very ill and findings were so transitory that no baseline for accurate evaluation could be obtained. From the time of first observation until the present there was a progressive improvement in the overall picture, so that any compound employed might have given the impression of being beneficial.”*—Chief Medical Officer Captain L.T. Coggeshall, 1946, after receiving evacuated U.S. Marines with filariasis infections from INDOPACOM during the Second World War.^1^

Filariasis is a nematode infection of humans spread by mosquitoes that is infamous for its end stage syndrome of elephantiasis or severe edema caused by lymphatic damage. Mass drug administration (various combinations of diethylcarbamazine, albendazole, and ivermectin) has greatly reduced the global burden of this neglected tropical disease, but the disfigurement of this severe chronic disease gives lymphatic filariasis an impact beyond its acute clinical symptoms. Realizing that some modern militaries (including the Australian Defence Force) were continuing to give presumptive anthelminthic treatment to soldiers returning from Melanesian deployments, the historical basis for this practice was investigated to determine its appropriateness. The military impact of lymphatic filariasis was largely limited to U.S. Army or Marine Corps units defending a few Pacific islands (Tonga, Samoa, Fiji) during the Second World War.^2^ Given the near absence of traveler infections in New Guinea and the greatly reduced worm burden in endemic populations, presumptive routine treatment for lymphatic filariasis should cease and treatment limited to those diagnosed with acute infections.

The U.S. military was aware of the risk of malaria during the Second World War campaign in the Southwest Pacific from its colonial history in the Philippines, but was much less prepared for the epidemic of lymphatic filariasis in a few island defence battalions in Polynesia starting in 1942.^2^ Sudden deployment of defensive forces ahead of what was feared to be further Japanese island invasions to cut Australian lines of sea communication with the U.S. resulted in inexperienced soldiers living in close proximity to the local Polynesian population. Epidemic lymphatic filariasis in the U.S. military was largely limited to islands such as Tonga and Samoa, with diurnally active parasites; few if any filarial infections resulted from the many soldiers sent to New Guinea, with nocturnally active parasites.^2^

Some soldiers developed acute symptoms such as lymphangitis, adenitis, and scrotal swelling weeks and months following exposure to *Wuchereria bancrofti*, which initially mystified medical officers who were both unfamiliar with tropical diseases and had no means of laboratory diagnosis. More than 10,000 cases of lymphatic filariasis are estimated to have resulted from less than 38,000 men exposed, of which 90% of cases occurred in a few U.S. Marine Corps island defense battalions.^1^

The major impact of the disease was psychological, related to the unknown cause and genital involvement among exclusively male military units isolated on Pacific islands far from any combat operations.^3^ No microfilaria were seen in the blood, and the few adult worms could not be demonstrated without direct pathological examination of lymph nodes. Great uncertainty prevailed among medical officers, which in turn promoted rumors among other service members. A few dramatic cases of elephantiasis among Polynesians increased concerns about the long-term effects of this unfamiliar tropical disease.

An administrative decision was enacted to remove sick men from the area until they could be diagnosed and treated, leading to the formation of several general hospitals in the U.S. specifically for tropical diseases. Most of those thought to have lymphatic filarial infections were sent to the U.S. Marine Corps Barracks in Klamath Falls, Oregon, which at its peak in 1944 had up to 2,000 inpatients (**Figure**).^4^ The senior medical officer at Klamath Falls, Captain L.T. Coggeshall, as the ninth Charles Franklin Craig Lecturer of the American Society of Tropical Medicine in 1944, stated:

“There is a very prominent psychic element in the picture, as could be expected with an infection that involved the genitalia plus the reaction after seeing the grotesque deformities in the natives with elephantiasis. Men are concerned about sterility and the possibility that continued assaults on the lymphatic system will leave a permanent lymph edema.”^4^

Treatment largely consisted of rehabilitation and sports, trying to break any association with the ‘sick role’.^5^ No chemotherapy was available or given, except for malaria relapses that occurred in some soldiers who had been deployed in the Solomon Islands.

Entire U.S. Army units were repatriated as combat ineffective due to lymphatic filariasis.^6,7,8^ Several hundred soldiers of the 134th Field Artillery and 404th Engineer Company were stationed on the main island of Tonga (Tongatabu or Tongtapu) from May 1942 until May 1943, when they were exposed to *Wuchereria bancrofti*.^2^ The soldiers began to develop symptoms—lymphangitis, scrotal swelling, inguinal adenopathy—after leaving Tonga, starting in August 1943, and were sent to an Army field hospital in New Guinea in January 1944. After being deployed for 27 months (7 of which were in hospital in New Guinea) those soldiers were then repatriated to Moore General Hospital in North Carolina in July 1944, through the end of the war. At no time did either unit participate in combat operations.^2^

Post-war syndromes are often difficult to evaluate, but now, decades later, it can be definitively stated that almost none of the feared long-term sequelae of lymphatic filariasis among U.S. soldiers occurred from infection during the Second World War.^9^ Only 1 case of elephantiasis was reported by a Veterans Administration hospital out of more than 10,000 symptomatic infections.^10^

Subsequent Indo-Pacific regional wars have demonstrated a very limited number of filarial infections in service members. During the French war in Indochina, 151 cases of filarial disease *Brugia malayi* were diagnosed in French soldiers in Algeria, following their return from Vietnam in 1951.^11^ The epidemic largely consisted of tropical eosinophilic pneumonia with adenopathy and pulmonary asthma. No microfilaria were seen in blood, and most soldiers either experienced spontaneous illness resolution or were treated with diethylcarbamazine without sequelae.

The U.S. Army in Vietnam had even fewer cases of lymphatic filarial infection described despite the deployment of more than 2 million U.S. soldiers and high infection rates in local Vietnamese defensive forces. Two case reports were found in the literature. In 1 case, a U.S. soldier with *Wuchereria bancrofti* microfilaria was observed after 11 months, and his adenopathy resolved on treatment.^12^ Another case was seen in a U.S. veteran with adenopathy and persistent eosinophilia that responded to diethylcarbamazine despite no microfilaria detection.^13^

Australian military cases of lymphatic filariasis in the Indo-Pacific Command (INDOPACOM) were difficult to associate with Pacific islands, as most cases from the World Wars were thought to be due to exposure as children in Queensland.^14^ Up to 10% of soldiers recruited from Queensland in the early 20th century were infected with filaria, based on a case series of 4,000 persons whose blood was examined in Southeast Queensland.^14^ Only 24 cases of lymphatic filarial infection (22 in Australia, 2 in New Guinea; <1:30,000) were diagnosed in Australian soldiers during the Second World War, despite high infection rates in local New Guinea populations.^15^

No Australian military cases of filariasis were noted during the Vietnam War. After 6 months' exposure in Timor Leste, some Australian soldiers had positive serological tests, most of which were thought to be due to exposure to dog filaria in Australia.^16^ There was no evidence of disease or symptoms attributable to filaria. The overall conclusion was that seroconversion after exposure for 6 months was unusual, and disease risk was nearly nonexistent.^16^

There have been no recognized infections attributed in decades to lymphatic filariasis in the Australian Defence Force. Current helminthic disease reported from the U.S. Department of Defense in 2021 agrees with this extremely low risk assessment.^17^ From 2012 to 2018, few (<40/year) tissue-invasive nematode infections were reported, and of those ascribed to filaria, few were confirmed cases based on laboratory testing. Similarly, travel medicine cases of lymphatic filariasis in civilians have been rare, usually only occurring in those living closely with local populations for extended periods of time.^18^

Although historical reviews state that the military needs to remain aware of the risk of lymphatic filarial infections in INDOPACOM, based on the U.S. Second World War experience in Polynesia, recent evidence of service member infection is vanishingly rare, and likely to remain so, due to the great decrease in disease burden from mass drug administration in Polynesia and Melanesia.^19^ Currently, the Australian Defence Force gives both albendazole and ivermectin to soldiers returning from Melanesian field exercises, whereas the New Zealand Defence Force and U.S. military forces do not. Although use of albendazole and ivermectin in millions of people reinforces the extremely favorable safety profiles of these medications, there seems to be little medical justification for their use in redeploying soldiers in INDOPACOM. Serosurveillance of the Papua New Guinea Defence Force, whose soldiers are exposed to filaria from birth, also show extremely low rates of infection. A 2019 survey of 235 soldiers from Papua New Guinea’s North Coast found no evidence of current filarial infection, based on microscopy and rapid diagnostic testing.^20^

Psychological aspects may still determine how one experiences worms or rates risk of infection, but given the current difficulties in compliance with any preventive medicine intervention, it seems best to eliminate preventive measures that cannot be justified with current data. Those living within endemic populations for extended periods such as Special Forces members may be exceptions, but use of prophylactic anti-filarial medications should no longer be part of health planning for modern military units in the INDOPACOM region.

## Figures and Tables

**Figure:**
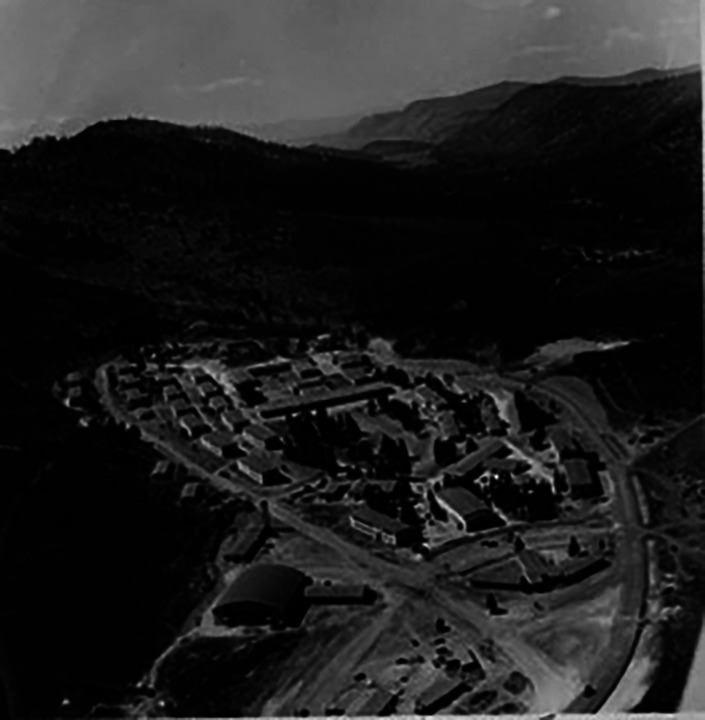
Marine Barracks, Klamath Falls, Oregon, circa 1944, Where Many U.S. Service Members Were Hospitalized for Filariasis After Infection in INDOPACOM

## AUTHOR AFFILIATIONS

Australian Defence Force Malaria and Infectious Disease Institute, Enoggera, Queensland: Dr. Shanks, Dr. Smith; University of Queensland, School of Public Health, Brisbane, Australia: Dr. Shanks; Metro North Hospital and Health Service, Queensland Health, Herston, Queensland, Australia: Dr. Smith

## References

[1] Coggeshall LT (1946). Filariasis in the serviceman: retrospect and prospect.. JAMA..

[2] Hayman JM Filariasis. In: Havens WP ed. Office of the Surgeon General. Internal Medicine in World War II: Volume III–Infectious Diseases and General Medicine. Department of the Army, U.S. Department of Defense 1968 123 144 Accessed Jul. 8, 2024. https://apps.dtic.mil/sti/pdfs/ADA292499.pdf

[3] Glauser F (1945). Filariasis in returning marines.. U.S. Naval Med Bull..

[4] Coggeshall LT (1945). Malaria and filariasis in the returning serviceman: the ninth Charles Franklin Craig Lecture.. Am J Trop Med..

[5] Winstead DK (1978). Filariasis bancrofti and chronic illness behavior.. Mil Med..

[6] Goodman AA, Weinberger E, Emmanuel M (1945). Studies of filariasis in soldiers evacuated from the South Pacific.. Ann Intern Med..

[7] Behm AW, Hayman JM (1946). The course of filariasis after removal from an endemic area.. Am J Med Sci..

[8] Swartzwelder JC Filariasis bancrofti. In: Coates JB Hoff EC eds. Office of the Surgeon General. Preventive Medicine in World War II. Department of the Army, U.S. Department of Defense 1964 63 72 Accessed Jul. 8, 2024. https://www.govinfo.gov/content/pkg/GOVPUB-D104-PURL-gpo87568/pdf/GOVPUB-D104-PURL-gpo87568.pdf

[9] Trent SC (1963). Reevaluation of World War II veterans with filariasis acquired in the South Pacific.. Am J Trop Med Hyg..

[10] Richman B, Young G (1995). Chronic elephantiasis: a case report.. J Am Podiatr Med Assoc..

[11] Galliard H (1957). Outbreak of filariasis (*Wuchereria malayi*) among French and North African servicemen in North Vietnam.. Bull World Health Organ..

[12] Colwell EJ (1970). Epidemiologic and serologic investigations of filariasis in indigenous populations and American soldiers in South Vietnam.. Am J Trop Med Hyg..

[13] Pittman FE (1972). Probable filariasis in a Vietnam veteran.. Am J Trop Med Hyg..

[14] Croll D (1919). Filariasis among Australian troops.. Br Med J..

[15] Walker AS Filariasis. In: Walker AS ed. Volume 1–Clinical Problems of War, Series 5–Medical, Australia in the War of 1939-1945. Australian War Memorial 1968 208 210 https://www.awm.gov.au/collection/C1417493

[16] Frances SP, Baade LM, Kubofcik J (2008). Seroconversion to filarial antigens in Australian Defence Force personnel in Timor-Leste.. Am J Trop Med Hyg..

[17] Lindrose AR, Mitra I, Fraser J, Mitre E, Hickey PW (2021). Helminth infections in the US military: from strongyloidiasis to schistosomiasis.. J Travel Med..

[18] Marcos LA, Shapley NP, Eberhard M (2014). Case report: testicular swelling due to lymphatic filariasis after brief travel to Haiti.. Am J Trop Med Hyg..

[19] Melrose WD, Leggat PA (2020). Acute lymphatic filariasis infection in United States Armed Forces personnel deployed to the Pacific area of operations during World War II provides important lessons for today.. Trop Med Infect Dis..

[20] McCallum F, Mond K, Cheng Q (2023). A health survey revealing prevalence of vector-borne diseases and tuberculosis in Papua New Guinea Defence Force personnel and families.. Am J Trop Med Hyg..

